# Tolerance and Physiological Correlates of Neuromuscular Electrical Stimulation in COPD: A Pilot Study

**DOI:** 10.1371/journal.pone.0094850

**Published:** 2014-05-09

**Authors:** Isabelle Vivodtzev, Benoit Rivard, Philippe Gagnon, Vincent Mainguy, Annie Dubé, Marthe Bélanger, Brigitte Jean, François Maltais

**Affiliations:** 1 Centre de Recherche, Institut Universitaire de Cardiologie et de Pneumologie de Québec, Université Laval, Québec, Canada; 2 Univ. Grenoble Alpes, HP2 (Hypoxia: respiratory and cardiovascular physiopathology), Grenoble, France; 3 Inserm HP2 (Hypoxia: respiratory and cardiovascular physiopathology), Grenoble, France; 4 Centre Hospitalier de Grenoble, Grenoble, France; 5 Département de physiothérapie, CHA-Hôpital de l'Enfant-Jésus, Québec, Canada; Pulmonary Research Institute at LungClinic Grosshansdorf, Germany

## Abstract

**Rationale:**

Neuromuscular electrical stimulation (NMES) of the lower limbs is an emerging training strategy in patients with COPD. The efficacy of this technique is related to the intensity of the stimulation that is applied during the training sessions. However, little is known about tolerance to stimulation current intensity and physiological factors that could determine it. Our goal was to find potential physiological predictors of the tolerance to increasing NMES stimulation intensity in patients with mild to severe COPD.

**Methods:**

20 patients with COPD (FEV_1_ = 54±14% pred.) completed 2 supervised NMES sessions followed by 5 self-directed sessions at home and one final supervised session. NMES was applied simultaneously to both quadriceps for 45 minutes, at a stimulation frequency of 50 Hz. Spirometry, body composition, muscle function and aerobic capacity were assessed at baseline. Cardiorespiratory responses, leg discomfort, muscle fatigue and markers of systemic inflammation were assessed during or after the last NMES session. Tolerance to NMES was quantified as the increase in current intensity from the initial to the final NMES session (ΔInt).

**Results:**

Mean ΔInt was 12±10 mA. FEV_1_, fat-free-mass, quadriceps strength, aerobic capacity and leg discomfort during the last NMES session positively correlated with ΔInt (r = 0.42 to 0.64, all p≤0.06) while post/pre NMES IL-6 ratio negatively correlated with ΔInt (r = −0.57, p = 0.001). FEV_1_, leg discomfort during last NMES session and post/pre IL-6 ratio to NMES were independent factors of variance in ΔInt (r^2^ = 0.72, p = 0.001).

**Conclusion:**

Lower tolerance to NMES was associated with increasing airflow obstruction, low tolerance to leg discomfort during NMES and the magnitude of the IL-6 response after NMES.

**Trial Registration:**

ClinicalTrials.gov NCT00809120

## Introduction

Neuromuscular electrical stimulation (NMES) of the lower limbs emerges as a useful alternative training strategy in patients with COPD [1]. In several controlled studies, this training technique is associated with improvements in quadriceps strength, exercise tolerance and quality of life [2],[3], [4],[5], [6], [7],[8].

The tolerance to NMES training, particularly the ability to tolerate progressively increasing current intensity, is an important determinant of the training response to this training modality [7]. For example, the increase in quadriceps strength and exercise tolerance following NMES training is related to the increase in current intensity during the training program [7], [8]. Although issues with tolerance to NMES training have received little attention, some evidence suggests that the ability to increase current intensity during the training program is highly variable across patients and is individual-specific [9], [10]. Learning about the reasons for the inter-individual variation in the tolerance to NMES training would be useful designing and selecting participants to NMES training intervention studies.

Several factors could be invoked for the interindividual variation in the tolerance to NMES. The magnitude of cardiac and ventilatory demands during NMES may be one of them. Depending on the intensity of the stimulation, up to a two-fold increase in resting cardiorespiratory parameters values may be expected [11]. Electrically-induced leg muscle fatigue [12] could increase leg discomfort during training and reduce tolerance to NMES. Subcutaneous body fat constitutes a resistance to electrical current in such a way that increasing current intensities may be more challenging in lean patients [13]. Finally, it is possible that the occurrence of a systemic inflammatory response following electrical stimulation could influence the tolerance to NMES but this has yet to be investigated. Based on these considerations, we hypothesized that high ventilatory and cardiac demands during NMES, poor aerobic capacity, the occurrence of muscle fatigue and leg discomfort during NMES, reduced fat-mass and the magnitude of the inflammatory response after NMES would be associated with poor tolerance to increasing current intensities during NMES training.

Accordingly, the aim of this study was to investigate the existing relationships between cardio-respiratory response during NMES, aerobic capacity, muscle fatigue, perception of leg discomfort during NMES, body composition, as well as systemic inflammation induced by NMES and the tolerance to NMES as defined by the increase in current intensity over the course of eight NMES training sessions.

## Methods

The protocol for this trial and supporting CONSORT checklist are available as supporting information; see Checklist S1 and Protocol S1.

### Study population

**Figure 1:**
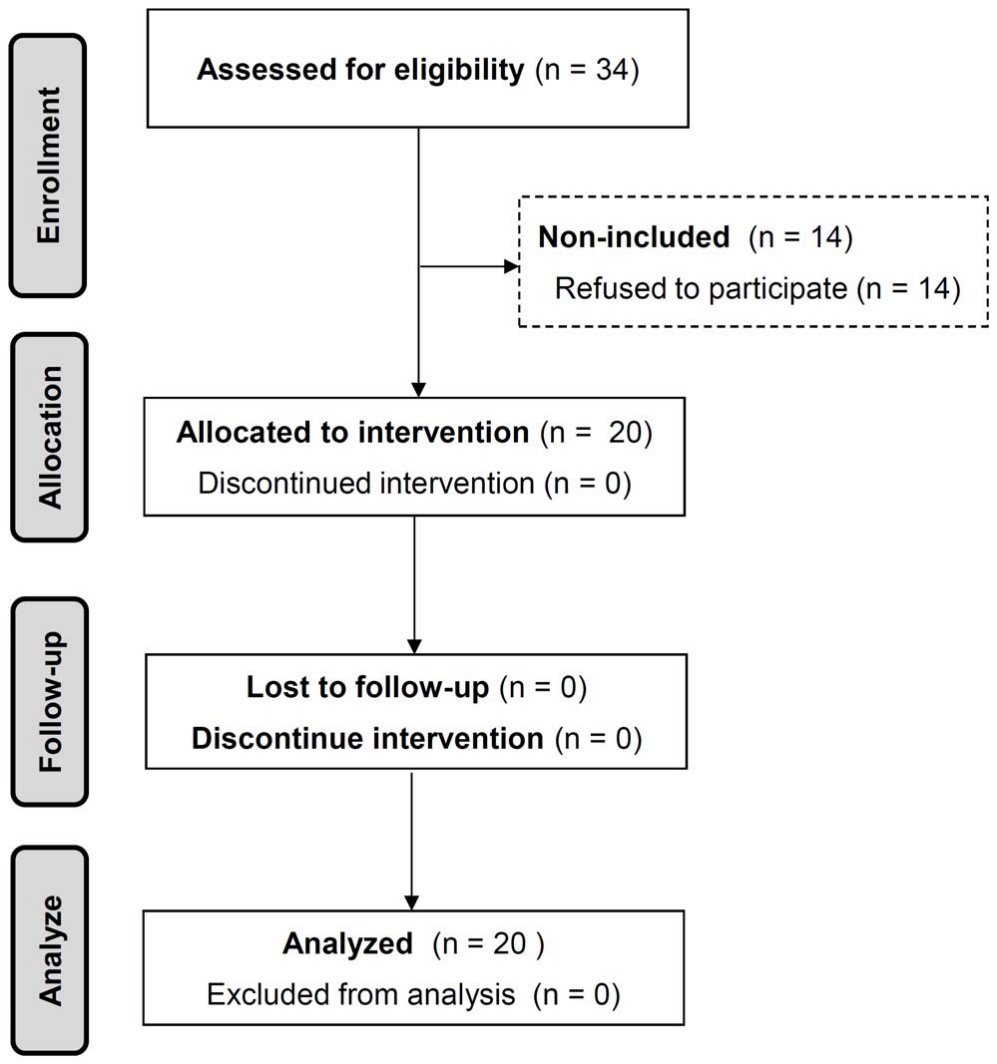
Study flow-chart.

The inclusion criteria of the study were: a) a diagnosis of COPD with a past or current smoking history >10 pack-year, b) age ≥40 years and c) able to read and write French or English. Patients presenting neuromuscular or vascular pathologies, or cutaneous disease of the legs preventing the application of the stimulation electrodes were excluded. The study flowchart is shown on figure 1.

### Study design

**Figure 2:**
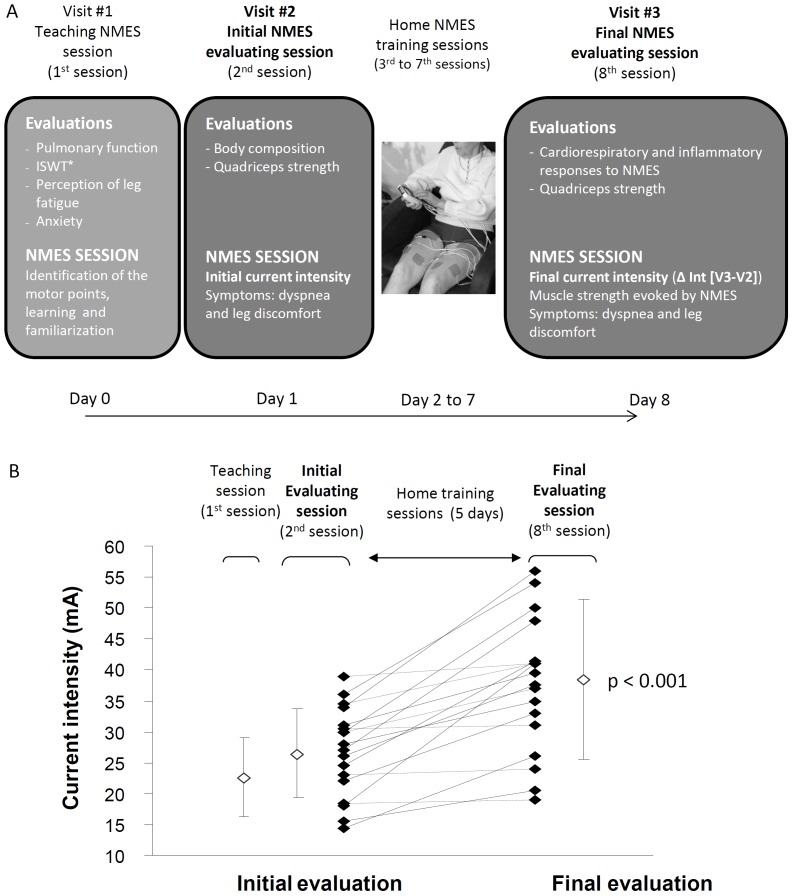
Study design and individual current intensities during NMES sessions. Panel A shows details and schedule of the 3 evaluations sessions. *Incremental shuttle walking test. Peak oxygen consumption (VO_2peak_) and perception of dyspnea and leg fatigue were assessed at end of ISWT (visit#1). Panel B shows the current intensities applied at the first session (teaching), at the second session (initial evaluation) session and at the 8^th^ session (final evaluation) (from left to right).

The study design is presented on figure 2A. Patients with mild to severe COPD (FEV_1_ = 56±14% predicted, body mass index = 27±5 kg/m^2^) first completed two supervised NMES sessions (one for teaching and one for evaluation), followed by 5 sessions at home for training and one final directly supervised session for evaluation, for a total of 8 sessions. Spirometry, body composition, muscle function and peak oxygen consumption (VO_2peak_) during an incremental shuttle walk and perception of leg discomfort during NMES were assessed at baseline (visit #1 or #2). Furthermore, the cardiorespiratory responses and perception of leg discomfort during NMES, muscle torque and muscle fatigue elicited by NMES as well as the intensity of the inflammatory response to NMES were measured during the final (8^th^) session (visit #3). Lastly, patients were asked before doing the first NMES session (Visit #1) whether they felt anxious or not and, after the last session (Visit #3), whether they would accept or not to use NMES training at home for muscle reinforcement. At the end of the study, the tolerance to NMES was quantified as the increase in stimulation current intensity from the second to final NMES training session (ΔInt). The study was conducted at the *Institut Universitaire de cardiologie et de pneumologie de Québec* and was approved by the ethic committee of this institution (CER20357). All patients gave written inform consent prior to study participation.

### Anthropometric data and spirometry

Height and weight were measured according to standardized methods [14]. Spirometry was obtained according to previously described guidelines [15] and related to the normal values of Quanjer and colleagues [16].

### Body composition

Dual energy X-ray absorptiometry (DEXA) provided regional assessment of fat and fat-free mass (General Electric Healthcare, Chicago, IL). Leg and whole-body fat-mass (FM) and fat-free-mass (FFM) were measured.

### Quadriceps strength and fatigue

Both voluntary and non-volitional muscle strength of the dominant quadriceps were measured before and after the final NMES session to assess muscle fatigue. Maximal voluntary contractions (MVC) of the quadriceps was measured during isometric contraction by a strain gauge (Hewlett-Packward), as routinely performed in our laboratory [17]. Potentiated quadriceps twitch tension (Twq) was measured by supramaximal magnetic stimulation of the femoral nerve (Magstim Co. Ltd., Whiteland, Wales, UK) using single twitch (1 Hz) but also paired twitch stimuli at 10 ms (100 Hz Twq, Twq_100_) and 100 ms (10 Hz Twq, Twq_10_) intervals, as previously reported in our laboratory [12]. Furthermore, the level of muscle torque elicited by NMES was measured during the final session and reported as percentage of MVC.

### Incremental shuttle walking test

Peak aerobic capacity was measured during the incremental shuttle walking test as previously described by Revill and colleagues [18] and used in our laboratory [19]. Cardio-pulmonary variables were monitored during exercise using a portable telemetric system (Jaeger Oxycon Mobile®, Germany). Resting and peak exercise values for heart rate (HR), oxygen consumption (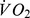) and ventilation (VE) were used as outcome parameters.

### Blood sampling and analysis

The antecubital venous blood was sampled between 8:00 a.m. and 10:00 a.m. in overnight-fasted patients, before, and immediately following the last NMES session. Plasma levels of systemic inflammatory (interleukin-6 [IL-6], c-reactive protein [CRP]) and oxidative stress markers (Advanced Oxidation Protein Products, AOPP) were measured as previously described in our laboratory [20].

### NMES training protocol

Motor points of the *vastus lateralis* and *medialis* were identified during the first NMES session (visit #1) by scanning the skin surface with a stimulation pen electrode as previously described [21]. During this and the second session, patients were also trained to become autonomous in using the stimulator in preparation for the home NMES training sessions. NMES training consisted in simultaneous electrically-induced contractions of both quadriceps using an electrostimulator (CEFAR Rehab4pro, Medical AB, Sweden) with the following parameters: 50-Hz frequency, 400 µs pulse duration and 7/10 seconds duty cycle (2 sec. ramp-up/1 sec. ramp-down). NMES was applied on the skin surface of upper and lower thigh using standard electrodes (50×50 cm and 90×50 cm for lower and upper thigh, respectively). Each session lasted 45 minutes. The current intensity was progressively increased during each session according to the patient's tolerance with the objective of reaching the highest tolerable current intensity. Leg discomfort was quantified using a 10-point modified Borg scale.

### Data analysis

The primary endpoint was the tolerance to NMES as defined by the increase in stimulation current intensity from the second to final NMES training session (ΔInt). Variables potentially influencing tolerance to NMES were grouped into five categories (Table 1): *i*) age and body composition, *ii*) pulmonary function, *iii*) exercise capacity and quadriceps strength *iv*) physiological response induced by NMES session (cardio-respiratory, muscle and inflammatory response to the last NMES session), and *v*) tolerance to leg discomfort (subjective perception of leg discomfort during NMES and anxiety).

**Table 1:** Variables potentially influencing tolerance to NMES.

Categories	Variables
**Body composition**	**Age**
	**Anthropometrics**: body mass index
	**Body composition**: leg and whole-body FM and FFM
**Pulmonary function**	**Airflow limitation**: FEV_1,_ FEV_1_/FVC, FVC
**Aerobic capacity and quadriceps strength**	**Peak oxygen consumption during ISWT**: 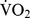 _peak_
	**Quadriceps strength**: MVC and Twq
**Physiological response to NMES**	**Cardiorespiratory response during NMES session** (HR, 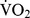 _,_ VE)
	**Quadriceps fatigue**: fall in Twq after NMES
	**Systemic inflammation** (IL-6, CRP) **and antioxidant** (AOPP)
**Personal sensibility**	**Symptoms at end of ISWT**: Dyspnea and leg fatigue
	**Symptoms during NMES**: Dyspnea and leg discomfort
	**Anxiety before** the 1^st^ session

Definitions of abbreviations: NMES: neuromuscular electrical stimulation; FM, whole-body fat mass; Leg FM, whole-body fat mass; FFM, whole-body fat-free-mass; leg FFM, Leg fat-free-mass; FEV_1,_ forced expiratory volume in 1 second; FVC: forced vital capacity; ISWT: incremental shuttle walking test; 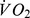: oxygen consumption; MVC, Maximal voluntary contraction; Twq: quadriceps twitch force; HR: heart rate; VE: minute ventilation; IL-6: Interleukin 6; CRP: C-reactive protein; AOPP: Advanced Oxidation Protein Products.

### Statistical analysis

Results are reported as mean ± SD. The normality of the variables was checked by the Kolmogorov-Smirnoff test. Muscle strength and inflammatory markers before and after the final NMES session were compared using paired-t-tests or Wilcoxon tests, depending on distribution normality. Cardiorespiratory parameters obtained at rest, at peak exercise and at the end of the final NMES session were compared with a single factor repeated measure of ANOVA with post-hoc paired-t-test using Bonferonni correction (or Holm-Sidak method when normality failed). Univariate correlation analyses were done using Spearman's correlation to identify potential determinants of the tolerance to NMES, as assessed by ΔInt. The independent variables that were associated with ΔInt with a p value<0.10 were included in a multiple linear regression model. Sigmastat (Dundas Software, Germany) was used for statistical analysis. Statistical significance was set at p<0.05.

## Results

### Patient characteristics at baseline

Among the thirty-four patients assessed for eligibility, twenty patients accepted to participate to the study. All of them completed the study and there was no drop-out during the training sessions (Figure 1). Baseline values for anthropometry, body composition, pulmonary function, inflammatory status, aerobic capacity are presented in Table 2. Patients presented a range of forced expiratory volume in 1 second (FEV_1_), and, on average, exhibited moderately severe airflow obstruction and exercise limitation with a peak 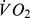
of 18 ml/kg/min.

**Table 2:** Subject characteristics.

Variables	n = 20
Gender (M/F)	14/6
Age (years)	65±5
BMI (kg/m^2^)	27±5
***Body composition***	
FFM (kg)	48±11
FFMI (kg/m^2^)	17±3
Leg FFM (kg)	16±4
FM (kg)	25±10
FM (% total body mass)	33±12
Leg FM (kg)	8±4
***Pulmonary function***	
FEV_1_ (L)	1.51±0.41
FEV_1_ (%)	54±14
FVC (L)	3.65±1.12
FEV_1_/FVC (%)	43±11
GOLD I, II, III, IV (n)	(1, 13, 5, 1)
***Peak ISWT Exercise Data***	
Walking distance (m)	366±146
Peak 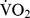 (ml/min/kg)	18±4
Dyspnea (Borg scale/10)	6.9±7.8
Leg fatigue (Borg scale/10)	5.3±1.6
	

Values are mean ± SD. Definitions of abbreviations: BMI: body mass index, FFM: Fat-free-mass, FFMI: fat-free-mass index, FM: Fat-mass, FEV_1_: forced expiratory volume in 1 s, FVC: forced vital capacity, ISWT: incremental shuttle walking test, 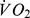: oxygen consumption.

### Tolerance to NMES training (ΔInt)

At the second session, the mean NMES current intensity was 27±7 mA; this corresponded to 144±27% of the minimal current intensity needed to induce perceptible muscle contractions. All patients completed the eight sessions. Anxiety before beginning the first session was reported in 45% of the patients although 75% of them subsequently stated they would accept this training strategy for further use at home. The mean increase in current intensity from the second to final NMES session (ΔInt) was +12±10 mA (range = 0.5 to 41 mA, p<0.001)(Figure 2B). Four out of the 20 patients were unable to increase current intensity. The maximal current intensity applied at the final session correlated significantly with the force of the quadriceps contraction elicited by NMES expressed in %MVC (r = 0.77, p<0.001) as well as with VO_2_ at the end of the final NMES session (r = 0.77, p≤0.002), suggesting that the strength of the resulting quadriceps contractions and metabolic demand elicited by NMES was dependent upon the stimulation current intensity. The perception of leg discomfort averaged 4.6±1.1 and 5.4±1.5 at end of the second and last NMES session, respectively.

### Physiological response to NMES session

#### Muscle fatigue induced by the final NMES session

NMES led to significant quadriceps fatigue as assessed by volitional (MVC) and non-volitional contractions (Table 3). Twq_,_ Twq_10_ and Twq_100_ were all significantly reduced after the final NMES session although highest fatigue was reported at 10 Hz. As a result, the Twq10/100 Hz ratio was reduced after NMES, suggesting that NMES mainly induced low-frequency fatigue of the quadriceps [22]. This amount of fatigue was reached for a mean torque developed during the final NMES session that corresponded to 9±7% of MVC.

**Table 3:** Muscle fatigue induced by NMES (at the 8^th^ session).

Variables	Pre NMES	Post NMES	Fall (% pre NMES value)	Post vs. pre p value
MVC	33.7±8.7	30.9±7.8	−8±8	<0.001
Single Twq	6.5±2.5	6.0±2.3	−14±17	0.017
10 Hz Twq	8.4±2.6	7.2±2.4	−16±14	<0.001
100 Hz Twq	13.7±3.6	12.5.±3.5	−9±13	0.003
Ratio 10/100 Hz Twq	0.62±0.10	±0.10	−7±10	0.021

Values are mean ± SD. Definitions of abbreviations: NMES: neuromuscular electrical stimulation; MVC, maximal voluntary contraction; Twq, quadriceps twitch tension; 10 Hz, 100 Hz are the frequencies used for paired Twq stimulation, representing 100 and 10 ms stimulating intervals, respectively, between the two stimuli.

#### Cardiorespiratory response during the final NMES session

Cardiac and ventilatory measurements during the final NMES session were not significantly different from resting values indicating that NMES did not induce important metabolic demands (Table 4). Despite the absence of cardiac and ventilatory stimulation, the perception of leg discomfort was similar at the end of the final NMES session and at peak ISWT.

**Table 4:** Cardiorespiratory responses at rest, at the end of the 8^th^ NMES session and at peak ISWT.

Variables	Rest	NMES (end of the 8^th^ session)	Peak ISWT	Rest vs. NMES p value
HR (bpm)	75±10	79±8	128±17*	0.45
VO_2_ (l/min)	0.26±0.06	0.30±0.07	1.37±0.41*	0.43
VE (L/min)	11.8±2.5	13.6±2.9	47.8±12.0*	0.21
SpO_2_ (%)	94±4	95±3	91±4*	0.89
Dyspnea (Borg)	0.4±0.6	0.6±0.5	6.9±7.8*	0.50
Leg discomfort (Borg)	1.1±2.4	5.4±1.5^†^	5.4±1.6^†^	<0.001

Values are mean ± SD. Definitions of abbreviations: NMES; neuromuscular electrical stimulation; ISWT, incremental shuttle walking test; HR, Heart rate; 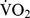
_:_ oxygen consumption; 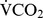
_:_ carbon dioxide output; RER, respiratory exchange ratio; VE, minute ventilation; SpO_2_, pulsed oxygen saturation. *Significantly different from rest and NMES. ^†^Significantly different from rest.

#### Inflammatory response after the final NMES session

With the exception of one patient who exhibited elevated IL-6 and CRP values (14 ng/L and 19 ng/L for IL-6 and CRP, respectively), the plasma levels of inflammatory markers were within normal values in study subjects (Table 5). After the final NMES session, IL-6 was increased in 13 patients although it was decreased in 7 patients. The mean post/pre NMES IL-6 ratio increased significantly from 3.3±2.7 to 3.6±3.1 pg/mL corresponding to 107±18% resting value (p = 0.02). There was no significant change in CRP and AOPP after NMES.

**Table 5:** Inflammatory response to NMES.

Variables	Pre NMES	Post NMES	Post/pre ratio	Post/pre p value
IL-6 (pg/mL)	3.3±2.7	3.6±3.1	1.07±0.18	0.029
CRP (mg/L)	3.5±4.0	3.7±4.9	1.01±0.09	0.198
AOPP (AU)	27.9±13.0	25.0±11.4	0.94±0.21	0.185

Values are mean ± SD. Markers of systemic inflammation: IL-6, interleukin 6 and CRP, C-reactive protein. Marker of oxidative stress: AOPP, Advanced Oxidation Protein Products. AU, arbitrary units in Chloramine-T equivalent.

### Physiological determinants of the tolerance to NMES (ΔInt)

**Figure 3:**
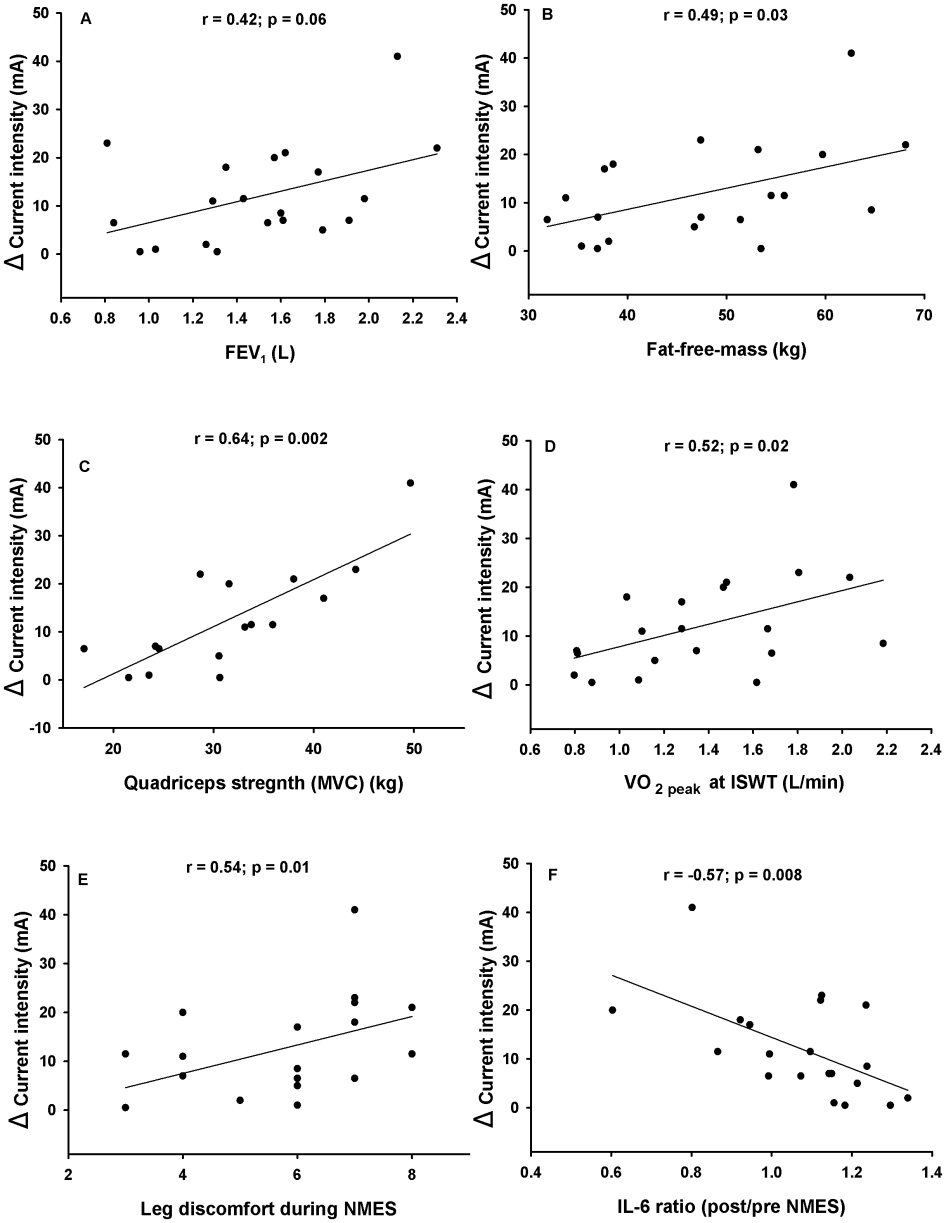
Relationships between tolerance to NMES (ΔInt) and its physiological correlates: (A) forced expiratory volume in 1 second (FEV_1_), (B) fat-free mass, (C) quadriceps strength, (D) VO_2peak_ at end of the incremental shuttle walk (ISWT) (E) the perception of leg discomfort during NMES and (F) the post/pre NMES plasma IL-6 ratio during the final NMES session. Spearman correlation coefficients are provided.

ΔInt correlated positively with FEV_1_ (r = 0.42, p = 0.06), fat-free-mass (r = 0.49, p = 0.03), quadriceps strength (r = 0.64, p = 0.001), peak 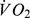
(r = 0.47, p = 0.03), perception of leg discomfort during the final NMES session (r = 0.54, p = 0.01), and negatively with post/pre NMES IL-6 ratio (r = −0.57, p = 0.008)(Figure 3). The extent of muscle fatigue, ventilation and heart rate responses at the final NMES session was not significantly associated with ΔInt. The same was true for age (r = 0.18, p = 0.42), body mass index (r = 0. 27, p = 0.23), fat mass (r = 0.10, p = 0.67) and perception of leg discomfort at the second NMES session (r = 0.33, p = 0.15).

In the multiple regression analysis, FEV_1_, leg discomfort during the final NMES session and the post/pre NMES IL-6 ratio were independently associated with ΔInt (cumulative r^2^ = 0.72, p<0.001)(Table 6).

**Table 6:** Predictors of the increase in current intensity from the second to the final NMES session (ΔInt) in multiple regression analysis.

Variables	cumulative r^2^	p value
FEV_1_	0.163	0.038
Whole-body FFM	-	0.37
MVC	-	0.58
VO_2peak_	-	0.40
Leg discomfort during NMES	0.289	0.001
post/pre NMES IL-6 Ratio	0.265	0.002
**Total r^2^**	0.719	0.001

Definitions of abbreviations: FEV_1_: forced expiratory volume in 1 s; FFM; Fat-free-mass; MVC, maximal voluntary contraction; VO_2_, oxygen consumption.

## Discussion

The present study aimed at investigating, in a detailed fashion, how patients with COPD tolerate NMES at its initiation. The main findings are that the tolerance to this intervention is variable from one patient to the other. We also explored predictors of the tolerance to NMES. Pulmonary function, reduced tolerability to leg discomfort during NMES and the IL-6 response following a NMES session were associated with reduced tolerance to the intervention. Muscle fatigue and cardio-respiratory responses to NMES were not associated with the ability to increase current intensity. Altogether, physiological variables that were assessed in the present study explained 72% of the variability in tolerance to increasing current intensity during NMES. Our investigation is clinically relevant in pointing out that not all patients with COPD tolerate well NMES and that those with advanced disease and poor tolerance to leg discomfort should be closely supervised at the beginning of NMES training program.

### Tolerance to NMES training in COPD

We and other have reported that increasing stimulation intensity during NMES training is crucial to the success of the intervention in terms of improving muscle function and functional capacity [7], [8]. In one study, patients who could not increase NMES current intensity by more than 10 mA or could not reach 30 mA of current intensity during a 6-week program did not exhibit improvement in muscle function and exercise tolerance following NMES [7]. One clinical implication of this is the necessity of helping patients to increase current intensity during NMES training. In the present study, while all patients started training with a perceptible contraction during NMES, a number of them doubled the current intensity from the first to the last session while others were unable to improve current intensity during the same period (Figure 2B). These less tolerant patients should be targeted for a closely supervised program to ensure that they can achieve sufficient current intensity during NMES sessions.

### Physiological determinants of the tolerance to NMES

#### Severity of the disease

ΔInt (+12±11 mA) after one week of NMES training was close to that previously achieved by patients with severe COPD after 6 weeks of training (+11 mA) [7]. In patients with mild COPD, a markedly higher current intensity (55 mA up to 100 mA) can be applied and tolerated at end of training [2], [6]. This basic observation that pulmonary function may influence the tolerance to NMES current intensity is strengthened by the relationship we found between ΔInt and FEV_1_ in the univariate and multiple regression analyses (Figure 3A and Table 6). We cannot provide a definitive answer as to why patients with more severe disease may be less tolerant to NMES, but the associations between ΔInt, fat-free-mass, muscle strength and aerobic capacity (Figure 3B, C and D) suggest that better preserved individuals are more tolerant to NMES perhaps because of a better tolerance to the discomfort related to progressively higher current intensities. This mechanism has been put forward to explain why some patients with COPD are more tolerant to high intensity exercise than others [23], [24]. In fact, the degree of acceptance to leg discomfort was one of the main contributors to the variance in the tolerance to NMES (Table 6, Figure 3E).

#### Muscle fatigue induced by NMES

NMES can induce muscle fatigue in healthy subjects [25] and patients with COPD [12] but this was observed when the stimulation intensity was determined by the investigator. In the present study, we confirmed the existence of quadriceps fatigue after the last NMES training session, using stimulation intensities that were self-determined by patients. Using paired magnetic twitch stimuli, we found that low-frequency-fatigue predominantly occurred after 10 Hz magnetic stimulation (Table 3), suggesting that contractile rather than neural properties were altered after NMES (low frequency fatigue), similarly to previous findings in healthy human [22]. This result could constitute a rationale for further investigation on the nature of muscle fatigue induced by NMES training. However, our findings suggest that muscle fatigue does not influence tolerance to NMES.

#### Cardio-respiratory response to NMES

In contrast to healthy subjects [11], we did not report significant changes in cardiorespiratory response during NMES as compared to resting conditions in patients with COPD (Table 4). This result confirms that NMES elicits only minimally the cardiac and respiratoy systems [26] and this was consistent with the low torque developed during the final NMES session (<10%MVC for 80% of the patients). Despite that, the perception of leg fatigue was similarly elevated during NMES and after ISWT, confirming the advantage of NMES in providing local muscle stimulations at a low cost for the cardio-respiratory systems.

#### Systemic inflammation induced by NMES

Although we recently reported that systemic inflammation did not worsen after 6 weeks of NMES training [7], an acute inflammatory response may still occur after one NMES session (Figure 3F). In the present study, we reported an increase in plasma IL-6 levels in 13 out of 20 patients after one NMES session (Table 5). This observation is consistent with rodent and human studies showing that repeated muscle contractions induced by electrical current [27] or during whole body exercise [28] is associated with elevated IL-6. We can only speculate on why an elevation in the IL-6 response to NMES could be a predictor of poor tolerance to this intervention. One possibility is that a pre-exercise glycogen depleted status in some patients might have influenced simultaneously the tolerance to NMES and the IL-6 response [29]. In addition to influencing the tolerance to NMES, an exaggerated inflammatory response could also prevent gains in muscle mass and strength following this intervention by impacting on signaling pathways involved in muscle mass regulation in COPD [30]. The link between inflammation and response to NMES is an area that should be further investigated.

### Potential limitations

We acknowledge that the correlation analysis may have suffered from relatively small sample size. Nevertheless, one strength of the present study is that patients were evaluated after familiarization with NMES at home, a situation that is informative for the design of home NMES training program. We recognize that non-physiological factors (i.e. anxiety, depression) potentially influencing tolerance to NMES were not exhaustively assessed. However, the fact that the multiple regression analysis could explain almost three quarter of the variation in the tolerance to NMES indicate that frail patients with advanced disease and poor tolerance to leg discomfort during NMES should be closely monitored to assess their tolerance to NMES. We elected to evaluate patients after 8 sessions (one week) of NMES training. This duration was selected for pragmatic reasons considering that if the intervention is poorly tolerated, it would still be timely to consider implementing strategies to facilitate tolerance to the intervention while not compromising the whole program. A one-week duration of training was also chosen because we have learned in a previous 6-week NMES intervention study that patients who are poorly tolerant to NMES after the first week of treatment are unlikely to behave differently during the remainder of the program [7].

### Conclusion

NMES training was heterogeneously tolerated in patients with COPD. Some patients were unable to increase stimulation current intensity along the 8 training sessions, potentially compromising the effectiveness of the intervention. Pulmonary function, the tolerance to the leg discomfort during NMES and the IL-6 response to NMES collectively explained up to 72% of the variability in the tolerance to NMES. Considering the importance of increasing current intensity for the outcome of the NMES intervention, strategies such as direct physiotherapeutic assistance, simultaneous volitional contractions during NMES, novel NMES parameters or even therapeutic combinations should be further investigated to optimize the benefits of NMES training.

## Supporting Information

Checklist S1 **The CONSORT statement checklist of the study is reported here.**
(PDF)

Protocol S1 **The protocol of the study.**
(DOCX)
