# Supplementary material for: Tolerance and Physiological Correlates of Neuromuscular Electrical Stimulation in COPD: A Pilot Study
Source: PLoS One. 2014 May 9;9(5):e94850. doi: 10.1371/journal.pone.0094850 (PMC4015894; doi:10.1371/journal.pone.0094850)
Supplement: Protocol S1 — The protocol of the study. (DOCX) [file pone.0094850.s002.docx]

## TOES PROTOCOL: A STUDY OF TOLERANCE TO ELECTRICAL MUSCLE STIMULATION IN COPD PATIENTS

A study of the clinical tolerance, the cardio-respiratory response and the muscular fatigue induced by a session of electrical muscular stimulation in COPD patients

Authors: Isabelle Vivodtzev

François Maltais

Pierre LeBlanc

Yves Lacasse

Julie Milot

1. **Introduction**

Chronic Obstructive Pulmonary Disease (COPD) is a major cause of morbidity and mortality around the world and is associated with high health costs. Patient death is not solely due to loss of respiratory function but extra-pulmonary pathologies such as cardiovascular disease [1], osteoporosis, and depression [3] also contribute to the overall picture of the disease.

One systemic effect, muscular dysfunction is a key consequence of COPD. We now know that muscular weakness is associated with shorter survival, increased medico-economic costs [4, 5] and is a negative prognostic marker independent of the degree of respiratory malfunction [6]. Muscular dysfunction also contributes to a poor functional status [7] and a lower quality of life for these patients [8].

Rehabilitation to exercise is nowadays recognized as the best treatment modality able to improve functional ability to exercise and the quality of life of patients presenting moderate to severe COPD [9]. However, an improvement in aerobic fitness, which governs an individual’s fitness [10], does not have much effect on improving muscular weakness and muscular atrophy. A number of authors have therefore recommended that more emphasize is given to rebuilding muscular strength. Amongst the modalities of muscular rehabilitation, the localised exercise of muscles by electrical stimulation has been studied in COPD patients by several authors [11-15]. These studies show that this type of training has benefits for COPD patients, in terms of strengthening muscles, increasing functional abilities and improving quality of life, including for some COPD patients with chronic respiratory failure.

Electrical muscle stimulation has the advantage of specifically and locally targeting a chosen muscular group. Furthermore, the apparatus is light and manageable, easy to transport and can be used by the patient by-himself. Above all, the use of electrical muscle stimulation for patients with moderate to severe COPD is based on the hypothesis that it requires minimal respiratory effort while being carried out. Electrical muscle stimulation therefore seems to be an ideal therapy for the rehabilitation of patients who suffer from severe respiratory difficulties when doing physical exercise [16].

Nevertheless, our experience (ESTIM protocol, CER #20105) shows that some patients experience difficulties in increasing the intensity of stimulation, even after several weeks of training thus limiting the treatment’s effect [17]. However, the cardio-respiratory response of a COPD patient during a session of electrical muscle stimulation has never been described. Theurel’s study, carried out with healthy subjects, suggests that minute ventilation increases significantly more when the intensity of the electric stimulation is increased than during voluntary exercise of equal intensity. Minute ventilation may increase to 10L/min (23 ± 4 vs 13 ± 3 L/min at rest) when the intensity reaches 50% of the maximal voluntary contraction [18]. Furthermore, this study shows that oxygen consumption and heart rate increase with time and intensity during a session of electrical muscle stimulation. These factors could limit the ability for patients to tolerate electrical muscle stimulation in the same way as a decrease in oxygen saturation, and are worth investigating. What is more, the abilities to produce and to tolerate muscular contraction at a given stimulation intensity vary greatly for one individual and between individuals [19, 20] particularly for thinner individuals [14]. Body composition, especially at the site of stimulation, could influence the transmission of the electric stimulation and therefore could be partly responsible for intolerance to electrical muscle stimulation. Finally, there is currently no data concerning induced muscular fatigue and the metabolic expenditure associated with a complete session of electrical muscle stimulation in COPD patients. This type of information could help determine the minimum delay to respect between two sessions of electrical muscle stimulation in order to allow optimal muscular recovery in patients and improve long-term tolerance to a course of electrical muscle stimulation.

We therefore propose to evaluate COPD patient tolerance to electrical muscle stimulation together with the cardio-respiratory response, the symptoms linked to exercise (daily exercise, maximal exercise) and felt during the electrical muscle stimulation itself (pain perception), body composition, and the muscle fatigue which follows a stimulation session.

This study will allow us to describe the muscular and cardio-respiratory physiological responses associated with this type of training session with the aim of better adapting electrical muscle stimulation programmes to COPD patients according of their clinical tolerance.

**Objectives**:

**Overall objective:**

To study the tolerance of COPD patients to electrical muscle stimulation (EMS) (the ability to increase the intensity during one session and over the course of several sessions).

**Specific objectives:**

1. To determine whether an increase in ventilation, an acceleration in heart rate and/or a decrease in arterial oxygen saturation during electrical muscle stimulation is/are associated with COPD patient intolerance to EMS.
2. To determine whether pain perception influences COPD patient tolerance to electrical muscle stimulation and whether it changes over the course of several EMS sessions.
3. To determine whether body composition around the quadriceps influences COPD patient tolerance to electrical muscle stimulation.

**Secondary objectives:**

1. To quantify the muscular fatigue induced by a session of electrical muscle stimulation in COPD patients.
2. To determine whether electrical muscle stimulation increases systemic inflammation and oxidative stress.

**Hypotheses:**

**Main hypothesis:**

Some COPD patients have a low tolerance to electrical muscle stimulation (small or null increase in intensity after 5 sessions).

**Specific hypotheses:**

A large increase in ventilation, an acceleration in heart rate and/or a fall in arterial oxygen saturation during electrical muscle stimulation decreases patient tolerance to EMS.

Excessive pain perception lowers tolerance to electrical muscle stimulation. Pain perception diminishes over the course of several EMS sessions when electrical muscle stimulation is applied progressively.

Low body fat around the quadriceps is associated with increased pain perception during a session of electrical muscle stimulation and is therefore associated with lower tolerance to this training modality.

**Secondary hypotheses:**

Peripheral muscular strength is reduced following electrical muscle stimulation in COPD patients (muscular fatigue).

Systemic inflammation and oxidative stress are increased after electrical muscle stimulation.

Excessive muscular fatigue and/or the increased production of inflammatory proteins could contribute to a patient’s inability to increase EMS intensity over the course of several sessions and therefore limit the effectiveness of this training modality.

1. **Protocol**

**Inclusion criteria:**

Age ≥ 40 years

Patients presenting chronic obstructive pulmonary disease (COPD) (GOLD II to IV)

Smoker or ex-smoker (10 pack years or more)

Absence of any known neuromuscular pathology

Absence of any vascular pathology (legs especially)

Absence of any skin disorders on the legs

**Groups**:

All patients will receive the same evaluations and follow the same protocol.

We will include equal numbers of patients from different severity groups: GOLD II, III and IV.

**Sample size**

As this is an observational study, we will include 3 groups of 7 COPD patients with different severity (GOLD II, III and IV) giving a total of 21 patients.

**Study schedule**

Participation in this study involves 3 visits. The first and second visits (V1 and V2) will be separated by a day while the third visit (V3) will occur 7 days after V2. During these 7 days, the patient will be asked to carry out 5 sessions of electrical muscle stimulation training by himself, spread out as whenever he likes. He will be given the electrical stimulator and the electrodes during V2.

**V1: Baseline assessment and familiarisation with EMS**

During the first visit, the consent form, a spirometry test and a bleep fitness test will be completed. The aim of this session will also be for the patient to familiarise himself with the apparatus (how to use the electrical stimulator, how to stick the electrodes to the skin, how to control the stimulation intensity).

**V2: Autonomous use of EMS, measure of pain perceived and of muscle fatigue**

The aim of this visit is to ensure that the patient can carry out electrical muscle stimulation in autonomy so that he will be able to use the apparatus at home. A full stimulation session will be carried out during which the patient will progressively take charge of the session. During electrical muscle stimulation, symptoms will be monitored (pain perception). The strength of the quadriceps will be tested before and after the session in order to measure the muscular fatigue caused by an initial EMS session. Finally, body composition will be determined during the course of this visit.

**V3: Assessment of EMS progression. Measure of pain perceived and of muscle fatigue at EMS intensity used during the sessions at home.**

The aim of this visit is to measure the pain perceived during a session of electrical muscle stimulation carried out at the EMS intensity that was used during the sessions at home. At the start of the session, the intensity of the stimulation applied will be that reached during visit 2, in order to compare the pain perceived for a given intensity, after 7 days of using the apparatus. Next, the patient will be asked to increase the intensity up to the level reached at home in order for us to calculate the intensity increase delta between V2 and V3. Throughout the course of this EMS session, the cardio-respiratory response will be measured using a portable “metabograph” fitted with a telemetric system (Oxycon mobile, Viasys). Furthermore, a blood sample will be taken before and after the session in order to measure systemic inflammation and oxidative stress levels. Lastly, muscular strength will be measured in order to determine the fatigue induced by this session.

**Measurements**

***Pulmonary function test***

Measurements of expiratory flow and inspiratory capacity will be carried out according to the ATS recommendations and performed by technicians trained in this type of tests.

***Blood samples***

A nurse, member of our research team, will take two blood samples during visit 2.

**Blood test #1 – before the EMS session:** (maximum volume = 20mL)

1. A complete blood count (4mL)
2. Inflammatory proteins and oxidative stress (12mL)
3. **CRP:** An Enzyme-Linked Immunoabsorbent Assay (ELISA) will be used to measure CRP (C Reactive Protein), an acute phase inflammatory protein.
4. **IL-6:** An Enzyme-Linked Immunoabsorbent Assay (ELISA) will be used to measure IL-6 (Interleukin-6), an inflammatory cytokine produced by muscles and found at its highest levels during muscular contraction.
5. **AOPP:** Proteins are susceptible to damage by free radicals induced by oxidative stress. AOPP (Advanced Oxidation Protein Products) is monitored in the plasma and expressed as chloramine-T equivalents by measuring the absorbance in acidic conditions at a wavelength of 340 nm in the presence of potassium iodide.

**Blood test #2 – after the EMS session:** (volume = 12mL)

Inflammatory proteins and oxidative stress (12mL)

1. CRP
2. IL-6
3. AOPP

Blood will be centrifuged in order to separate the plasma and will be conserved at -80°C for subsequent analyses. If the patient accepts and signs the “Banque de Tissus Pneumologie” (Pulmonology Tissue Bank) form, the remaining blood will be banked.

***Pain perception***

Pain perception will be measured using a visual analogue scale (VAS) with one non-graduated side (for the patient) and one graduated side (for the investigator) used to put a number on the measure.

***Assessment of body composition using DEXA***

Body composition will be assessed using DEXA which allows the local quantification of bone density, lean body mass and body fat. This measure will allow us to determine the regional distribution of lean body mass vs body fat.

***Assessment of quadriceps strength and fatigue***

All measurements will be carried out on the dominant quadriceps muscle with the patient lying on his back, with the knee at a 90º angle, in a position standardised by Man et al. [21]. For the sake of the patient’s comfort, his back can be raised by an angle of 30º relative to the horizontal.

The battery of tests includes:

1. ***Maximum voluntary strength (MVS)***

Five measurements will be carried out allowing for a recovery period of 2 minutes between contractions. Measurements must be reproducible (<10% variation). The highest measurement, sustained for at least 1.5 seconds, will be used for analytical purposes. During a voluntary muscular contraction, fatigue of the rectus femoris and the vastus lateralis muscles will be objectified in real-time using surface electromyography (Biopac System) by means of electrodes placed on the skin near each muscular head. Three EMG electrodes will be placed at the heads of the quadriceps and the muscle’s electric activity will be monitored throughout the exercise.

1. ***Quadriceps twitch force (non-cooperative force) measurements***

The strength of the dominant quadriceps will also be assessed independently of the patient’s motivation during a supramaximal magnetic stimulation of the femoral nerve. This measurement will be carried out with the patient in the supine position, his knee and hip at 45º angles, as frequently performed in our lab [7]. We have previously validated this method, demonstrating its reproducibility and sensitivity to change [7].

***Bleep fitness test***

In order to quantify the patient’s cardio-respiratory response to maximum effort, patients will undergo an incremental bleep fitness test as frequently carried out in our lab (Pepin et al. 2005). This test uses a portable “metabograph” fitted with a telemetric system (Oxycon mobile, Viasys) to measure ventilation, oxygen consumption, heart rate and saturation throughout the exercise. Physiological values measured during a session of electrical muscle stimulation can therefore be compared to the maximal values measured during this test. Furthermore, this test will allow us to characterise the patients’ physical condition. Fifteen minutes before this test an electrocardiogram (ECG) at rest will be carried out.

## TOES PROTOCOL: A STUDY OF TOLERANCE TO ELECTRICAL MUSCLE STIMULATION (EMS) IN COPD PATIENTS


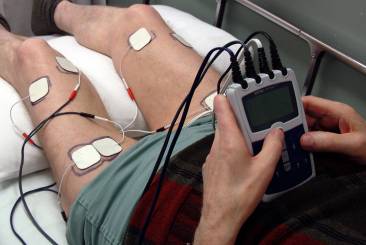


**Visit 3**: 3h

- Blood sample
- Muscle strength
- **ESM training session with mobile Oxycom** (cardio-respiratory monitoring of the exercise) (30 min)
- Blood sample
- Muscle strength

**Visit 2**: 3h

- Muscle strength
- **Session to gain autonomy of use with ESM** (30 min)
- Muscle strength
- DEXA

**Visit 1**: 3h

- Consent
- Spirometry
- Fitness test (with CI)
- **EMS initiation session** (10 -15 min)

**ESM at home**

5 sessions carried out in autonomy

Contact by phone

D0 + 8

D0 + 1

D0

**Details of the electrical muscle stimulation sessions**

General concept: Simultaneous muscular stimulation of both legs induced by an electric current conducted by two cutaneous electrodes. Throughout the session, a period of muscular contraction alternates with an “active” recovery period (stimulation frequency which favours local blood circulation). The stimulation intensity is progressively increased provided it is well tolerated by the patient.

Site: LAVAL hospital and at the patient’s home

**Visit 1: Initiation session:** presentation of the electrical muscle stimulation program, positioning of the electrodes and learning how to use the apparatus.

**Visit 2: Session to gain autonomy of use:** observation that the patient can satisfactorily carry out an EMS session autonomously.

**At home: five EMS sessions carried out autonomously at home**

During the seven days separating visits V2 and V3, the patient will be required to carry out 5 sessions at home. He will be asked to increase the EMS intensity as much as he can throughout one session and from one session to another, whilst staying beneath his pain threshold. In other words, he will have to increase the intensity frequently in order to always be at the maximum bearable intensity. For each session, the patient will have to write down the initial and final intensities along with the level of pain perceived for each. Throughout the week, we will maintain contact by phone (one initial phone call the day of the first session at home and then according to the patient’s needs) in order to answer any questions and/or solve any potential technical difficulties.

**Visit 3: Training session,** session as carried out by the patient at home.

Electrode placement

**Three electrodes on each leg:** two electrodes (50*50 mm) are placed on the skin by the lateral and the medial vastus of the quadriceps + one electrode (50*90 mm, double outlet) placed horizontally towards the top of the thigh, beneath the groin.

**
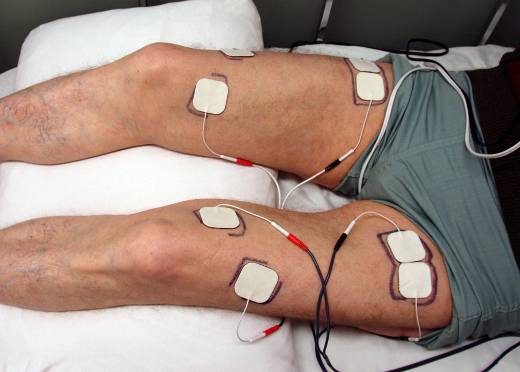

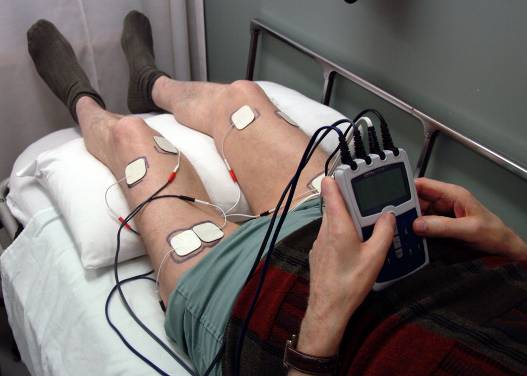
**

*Electrode placement for thigh rehabilitation*

*In this picture, the electrodes placed at the upper thigh are smaller models which will be replaced by electrodes of larger calibre, positioned side by side lengthwise (59*90 mm)*

**Electrode placement will be standardised** so as to lie by the lateral and the medial vastus motor points. The motor points will be identified for each patient individually, using a **pen electrode** that helps determine motor pathways and points of emergence.

Program

**Intermittent frequencies**: contraction at 50 Hz / “active” muscle relaxation at 5 Hz

- - - contraction 7 sec / relaxation 10 sec

**Time to reach contraction**: 3 seconds / time to relaxation: 1 second. Considering that the contraction is incomplete during the first two seconds of the contraction process, the cycles are 30%.

**Pulse width** = 400 µm

**The intensity** at which the patient begins to feel contraction of his thigh muscle is defined as the initial intensity; it is then progressively increased in order to reach the maximal bearable intensity.

**Patient recruitment**

The host laboratory (Laval Hospital) is a research centre that ensures a regular recruitment of patients to clinical research projects.

**Electrical muscle stimulation implementation**

The use of electrical muscle stimulation as a home-based rehabilitation protocol is currently under investigation as part of the ESTIM protocol (CER # 20105). The main purpose of this protocol is to describe patient tolerance to EMS (symptoms with effort) and the physiological effects induced by an EMS training session in the context of a rehabilitation program (during the 7^th^ session). In this protocol, electrical muscle stimulation is first used at the hospital, subsequently performed at home in five sessions spread over seven days, and finally used to assess the patient during a seventh personalised session.

**Criteria used for analysis**

- Tolerance to EMS: pain perceived for a given stimulation intensity and the increase in intensity between sessions V2 and V3, ie. ∆Intensity (V3-V2)

## Comparison of the cardio-respiratory values in response to EMS against the maximum physiological values recorded during the fitness test.

## Change in muscular function after the EMS session

## Change in plasma protein content after the EMS session

**Analysis of the results**

Using simple and multiple regressions, the relationship between the different variables will be analysed with the aims to:

**Objective #1**

Identify correlations between ∆Intensity (V3-V2) and the cardio-respiratory response to EMS (raw values and % of maximum values)

**Objective #2**

Identify correlations between ∆Intensity (V3-V2) and the pain perceived during the initial session V2 (VAS reading for the intensity reached during V2) and between ∆I (V3-V2) and the change in pain perceived between V2 and V3 (VAS reading at equal intensity)

**Objective #3**

Identify correlations between ∆Intensity (V3-V2) and body composition around the quadriceps

**Objective #4**

Identify correlations between ∆Intensity (V3-V2) and muscular fatigue induced by EMS.

**Objective #5**

Identify correlations between ∆Intensity (V3-V2) and the change in plasma protein content (CRP, IL-6 and AOPP) after EMS

**3. Future directions for the application and possible development of this project and potential effects on respiratory health.**

EMS is a method applicable to the rehabilitation of COPD patients but is still relatively unknown and under-employed. The investigator’s role is to supply accurate and reliable physiological data that could be used to better define patient tolerance to electrical muscular stimulation. What is more, a study such as the one described here, will allow the in-depth study of the mechanisms involved in muscular fatigue induced by EMS when performed in one of the best equipped centres with advanced understanding of the mechanisms of muscular function in COPD patients.

## 4. Bibliography

1. Sin, D.D. and S.F. Man, *Chronic obstructive pulmonary disease as a risk factor for cardiovascular morbidity and mortality.* Proc Am Thorac Soc, 2005. **2**(1): p. 8-11.

2. de Vries, F., et al., *Severity of obstructive airway disease and risk of osteoporotic fracture.* Eur Respir J, 2005. **25**(5): p. 879-84.

3. Brenes, G.A., *Anxiety and chronic obstructive pulmonary disease: prevalence, impact, and treatment.* Psychosom Med, 2003. **65**(6): p. 963-70.

4. Hamilton, A.L., et al., *Muscle strength, symptom intensity, and exercise capacity in patients with cardiorespiratory disorders.* Am J Respir Crit Care Med, 1995. **152**(6 Pt 1): p. 2021-31.

5. Decramer, M., et al., *Muscle weakness is related to utilization of health care resources in COPD patients.* Eur Respir J, 1997. **10**(2): p. 417-23.

6. Marquis, K., et al., *Midthigh muscle cross-sectional area is a better predictor of mortality than body mass index in patients with chronic obstructive pulmonary disease.* Am J Respir Crit Care Med, 2002. **166**(6): p. 809-13.

7. Saey, D., et al., *Contractile leg fatigue after cycle exercise: a factor limiting exercise in patients with chronic obstructive pulmonary disease.* Am J Respir Crit Care Med, 2003. **168**(4): p. 425-30.

8. Mostert, R., et al., *Tissue depletion and health related quality of life in patients with chronic obstructive pulmonary disease.* Respir Med, 2000. **94**: p. 859-867.

9. Lacasse, Y., et al., *Pulmonary rehabilitation for chronic obstructive pulmonary disease.* Cochrane Database Syst Rev, 2002(3): p. CD003793.

10. Casaburi, R., et al., *Physiologic benefits of exercise training in rehabilitation of patients with severe chronic obstructive pulmonary disease.* Am J Respir Crit Care Med, 1997. **155**(5): p. 1541-51.

11. Neder, J.A., et al., *Home based neuromuscular electrical stimulation as a new rehabilitative strategy for severely disabled patients with chronic obstructive pulmonary disease (COPD).* Thorax, 2002. **57**(4): p. 333-7.

12. Bourjeily-Habr, G., et al., *Randomised controlled trial of transcutaneous electrical muscle stimulation of the lower extremities in patients with chronic obstructive pulmonary disease.* Thorax, 2002. **57**(12): p. 1045-9.

13. Zanotti, E., et al., *Peripheral muscle strength training in bed-bound patients with COPD receiving mechanical ventilation: effect of electrical stimulation.* Chest, 2003. **124**(1): p. 292-6.

14. Vivodtzev, I., et al., *Improvement in quadriceps strength and dyspnea in daily tasks after 1 month of electrical stimulation in severely deconditioned and malnourished COPD.* Chest, 2006. **129**(6): p. 1540-8.

15. Dal Corso, S., et al., *Skeletal muscle structure and function in response to electrical stimulation in moderately impaired COPD patients.* Respir Med, 2007. **101**(6): p. 1236-43.

16. Vivodtzev, I., Y. Lacasse, and F. Maltais, *Neuromuscular electrical stimulation of the lower limbs in patients with chronic obstructive pulmonary disease.* J Cardiopulm Rehabil Prev, 2008. **28**(2): p. 79-91.

17. Lake, D.A., *Neuromuscular electrical stimulation. An overview and its application in the treatment of sports injuries.* Sports Med, 1992. **13**(5): p. 320-36.

18. Theurel, J., et al., *Differences in cardiorespiratory and neuromuscular responses between voluntary and stimulated contractions of the quadriceps femoris muscle.* Respir Physiol Neurobiol, 2007. **157**(2-3): p. 341-7.

19. Lloyd, T., et al., *A review of the use of electro-motor stimulation in human muscles.* Aust J Physiother, 1986. **32**: p. 18-30.

20. Kubiak, R.J., K.M. Whitman, and R.M. Johnston, *Changes in quadriceps femoris muscle strength using isometric exercise versus electrical stimulation.* J Orthop Sports Phys Ther, 1987. **8**: p. 537-541.

21. Man, W.D., et al., *Non-volitional assessment of skeletal muscle strength in patients with chronic obstructive pulmonary disease.* Thorax, 2003. **58**(8): p. 665-9.
